# *Pseudomonas fluorescens* group bacterial strains interact differently with pathogens during dual-species biofilm formation on stainless steel surfaces in milk

**DOI:** 10.3389/fmicb.2022.1053239

**Published:** 2022-10-26

**Authors:** Mehdi Zarei, Saeid Rahimi, Per Erik Joakim Saris, Amin Yousefvand

**Affiliations:** ^1^Department of Food Hygiene, Faculty of Veterinary Medicine, Shahid Chamran University of Ahvaz, Ahvaz, Iran; ^2^Department of Microbiology, Faculty of Agriculture and Forestry, University of Helsinki, Helsinki, Finland

**Keywords:** Biofilm, *Pseudomonas fluorescens*, *Staphylococcus aureus*, *Bacillus cereus*, *Escherichia coli* O157:H7, *Salmonella* Typhimurium

## Abstract

In order to develop strategies for preventing biofilm formation in the dairy industry, a deeper understanding of the interaction between different species during biofilm formation is necessary. Bacterial strains of the *P. fluorescens* group are known as the most important biofilm-formers on the surface of dairy processing equipment that may attract and/or shelter other spoilage or pathogenic bacteria. The present study used different strains of the *P. fluorescens* group as background microbiota of milk, and evaluated their interaction with *Staphylococcus aureus*, *Bacillus cereus*, *Escherichia coli* O157:H7, and *Salmonella* Typhimurium during dual-species biofilm formation on stainless steel surfaces. Two separate scenarios for dual-species biofilms were considered: concurrent inoculation of *Pseudomonas* and pathogen (CI), and delayed inoculation of pathogen to the pre-formed *Pseudomonas* biofilm (DI). The gram-positive pathogens used in this study did not form dual-species biofilms with *P. fluorescens* strains unless they were simultaneously inoculated with *Pseudomonas* strains. *E. coli* O157:H7 was able to form dual-species biofilms with all seven *P. fluorescens* group strains, both in concurrent (CI) and delayed (DI) inoculation. However, the percentage of contribution varied depending on the *P. fluorescens* strains and the inoculation scenario. *S. Typhimurium* contributed to biofilm formation with all seven *P. fluorescens* group strains under the CI scenario, with varying degrees of contribution. However, under the DI scenario, *S.* Typhimurium did not contribute to the biofilm formed by three of the seven *P. fluorescens* group strains. Overall, these are the first results to illustrate that the strains within the *P. fluorescens* group have significant differences in the formation of mono-or dual-species biofilms with pathogenic bacteria. Furthermore, the possibility of forming dual-species biofilms with pathogens depends on whether the pathogens form the biofilm simultaneously with the *P. fluorescens* group strains or whether these strains have already formed a biofilm.

## Introduction

More than 50 validly named species and a large number of unclassified isolates make up *Pseudomonas fluorescens* complex group, one of the most diverse groups within the *Pseudomonas* genus (Mulet et al., 2010; Gomila et al., 2015; Garrido-Sanz et al., 2016). There are nine subgroups within this complex group, including *P. fluorescens*, *P. jessenii*, *P. fragi*, *P. gessardii*, *P. corrugata*, *P. chlororaphis*, *P. mandelii*, *P. koreensis* and *P. protegens* (Garrido-Sanz et al., 2016). Bacterial strains of the *P. fluorescens* group are among the most important spoilage bacteria in milk and dairy products, and have been frequently isolated from fresh milk and cheese (Wang and Jayarao, 2001; De Jonghe et al., 2011; Martin et al., 2011; Carrascosa et al., 2015). Their ability to grow at low temperatures allows them to outgrow other bacteria in cold raw milk (Fricker et al., 2011; von Neubeck et al., 2015; Meng et al., 2017). Furthermore, *P. fluorescens* group strains have also been reported to cause repeat and sporadic post-pasteurization contamination, which results in shorter shelf life for pasteurized milk (Reichler et al., 2018).

*P. fluorescens* group bacterial strains are important in the dairy industry not only because of their heat-resistant enzymes, but also because of their ability to adhere to and from biofilms on the surface of milk tanks and other dairy processing equipment (Shpigel et al., 2015; Stoeckel et al., 2016). They are therefore able to withstand harsh conditions such as cleaning-in-place (CIP) processes, allowing them to remain within the dairy processing plant for extended periods of time (Cherif-Antar et al., 2016). Aside from contaminating subsequent batches of milk passing the biofilm region, *Pseudomonas* biofilms may also attract and/or provide shelter for other (spoilage or pathogenic) bacteria.

Biofilm formation by spoilage and pathogenic bacteria is a serious concern in the dairy industry, and hence has received much attention. A variety of different spoilage and pathogenic bacteria are known to attach to the internal stainless steel surfaces of the raw milk storage tanks and pipelines, where they can grow and form mono-or multi-species biofilms (Austin and Bergeron, 1995; Sharma and Anand, 2002; Latorre et al., 2010; Marchand et al., 2012; Anand et al., 2014; Cherif-Antar et al., 2016). Although biofilms in the dairy industry are more likely to be formed by spoilage bacteria (due to the higher population), pathogenic bacteria may also participate in the biofilm formation process, which may result in dual-or multi-species biofilms (Shi and Zhu, 2009; van Houdt and Michiels, 2010; Schirmer et al., 2013; Makovcova et al., 2017). In such cases, the cooperative interspecies interactions within dual-or multi-species biofilms are likely increase their resistance to adverse conditions compared to single-species biofilms. On the contrary, interspecific competition may occur and cause antagonistic effects (Yang et al., 2011; Elias and Banin, 2012; Rendueles and Ghigo, 2012; Liu et al., 2016).

To our knowledge, there is no information on the interaction between *P. fluorescens* group strains and bacterial pathogens from different genera during biofilm formation. Hence, in the present study, we used different strains of the *P. fluorescens* complex group as background microbiota of milk and evaluated their interaction with *Staphylococcus aureus*, *Bacillus cereus*, *Escherichia coli* O157:H7, and *Salmonella* Typhimurium during dual-species biofilm formation on stainless steel surfaces. We considered two separate scenarios for dual-species biofilms; (i) concurrent inoculation of *Pseudomonas* and the pathogen, and (ii) delayed inoculation of the pathogen to the pre-formed *Pseudomonas* biofilm.

## Materials and methods

### Bacterial strains and culture conditions

This study used *P. fluorescens* ATCC 13525, six other *P. fluorescens* group bacterial strains previously isolated from cold raw milk: *P. fluorescens* 5a, *P. fluorescens* 21c, *P. fluorescens* 68a, *P. veronii* 25d, *P. cedrina* 69a, and *P. simiae* 77a (Zarei et al., 2020). *S. aureus* (ATCC 25923), *B. cereus* (PTCC 1154), *E. coli* O157:H7 (ATCC 43895), and *S.* Typhimurium (ATCC 14028) were also used in this study. Stock cultures of bacterial strains were stored at-70°C in Tryptic Soy Broth (TSB; Merck, Germany) supplemented with 25% (v/v) sterile glycerol (Merck, Germany). Bacterial strains were first activated by two successive transfers in TSB at 30°C for 48 h. To prepare the inoculum, 0.1 ml of activated culture was added to 10 ml of ultra-high temperature (UHT) milk (3.79% protein and 1.5% fat), and incubated at 30°C for 48 h. Commercial UHT milk from the same batch was used for all experiments throughout the study.

### Biofilm formation on stainless steel surfaces

#### Single-species biofilm

To evaluate the ability of individual *Pseudomonas* strains and the pathogenic bacteria to produce a biofilm on stainless steel surfaces, overnight cultures of each strain were diluted to the final concentration of 10^6^ CFU/ml in UHT milk. The inoculated UHT milk (2 ml) was added to 12-well plates containing 1 × 1 cm stainless steel coupons (AISI 304, 2B, Norsk Stål AS, Norway), and incubated at 7°C for 48 h before quantifying the biofilm.

#### Dual-species biofilms

Each of the seven *Pseudomonas* strains was tested in dual-culture with each individual pathogen. The individual strain cultures were added to UHT milk and combined to contain approximately 10^6^ CFU/ml of each strain.

To investigate dual-species biofilms, two scenarios were considered. In the first scenario, an individual strain of *Pseudomonas* and the pathogen were inoculated concurrently (10^6^ CFU/ml of each strain) in UHT milk samples, and the formation of dual-species biofilms was evaluated. To achieve this, (hereinafter referred to as concurrent inoculation; CI), the combined dual-cultures in UHT milk (2 ml) were added to 12-well plates containing 1 × 1 cm stainless steel coupons, and incubated at 7°C for 48 h before quantifying the biofilm.

In the second scenario, the possibility of the pathogen being added to the pre-formed *Pseudomonas* biofilms was evaluated. For this scenario, (hereinafter referred to as delayed inoculation; DI), the UHT milk inoculated with 10^6^ CFU/ml of each individual *Pseudomonas* strain was added to 12-well plates containing stainless steel coupons, and incubated at 7°C. After 48 h of incubation, the wells were drained and replaced with 2 ml of UHT milk containing 10^6^ CFU/ml of each individual pathogen. The plates were then incubated for another 48 h before quantifying the biofilm.

#### Biofilm quantification

At the end of the incubation period, the wells were drained and the plates were gently washed three times by adding sterile dH_2_O (2 ml) to the coupon wells, followed by swirling of the plates and pipetting to remove non-attached bacteria. Biofilm cells were scraped into 1 ml of physiological saline solution using a cell scraper, and resuspended by vigorous pipetting for 15 s. Serial decimal dilutions of the cells were plated onto Tryptic Soy Agar (**TSA**) plates, and colonies were counted after 36 h of incubation at 30°C (Zarei et al., 2020).

#### Bacterial contribution to biofilm communities

To determine the percent contribution of bacterial strains to dual-species biofilms, all colonies within a zone of the TSA plates were selected and identified using KOH test, oxidase, and catalase tests, as shown in Table 1. The size of the zones for colony selection was adjusted to have approximately 30 colonies within the zone of TSA plates, with a total of 30–300 colonies (Heir et al., 2018).

**Table 1:** Identification key tests for differentiation of the colonies.

	Identification key		
Bacterial strain	KOH test	Catalase	Oxidase
*Pseudomonas* strains	+	+	+
*S. aureus*	−	+	−
*B. cereus*	−	+	+
*E. coli* O157:H7	+	+	−
*S.* Typhimurium	+	+	−

#### Statistical analysis

All experiments were replicated at least three times on different days. Results were analyzed using One-Way ANOVA (SPSS 20, SPSS Inc., Chicago, IL). The significance levels are expressed at a 95% confidence level (*p* ≤ 0.05) throughout.

## Results

### Single-species biofilms

**Figure 1:**
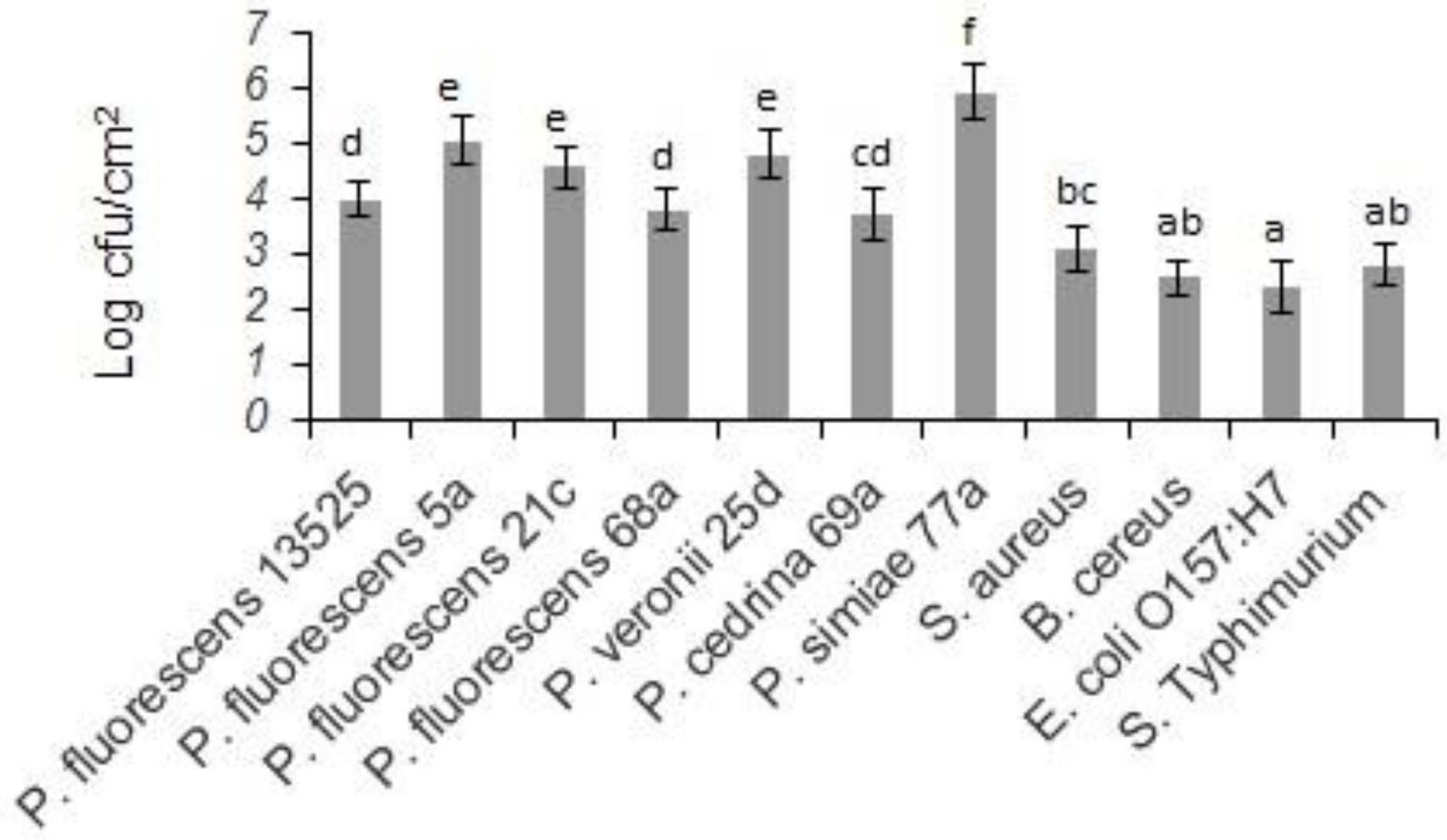
Biofilm cell counts of the selected *P. fluorescens* group strains and the pathogenic bacteria in mono-species biofilms on stainless steel surfaces after 48 h of incubation in UHT milk at 7°C. Different letters indicate significant differences between the strains.

Biofilm-forming ability of the selected strains of the *P. fluorescens* group and the pathogenic bacteria on stainless steel surfaces was evaluated in UHT milk. In general, after 48 h incubation at 7°C, the number of biofilm cells of the *Pseudomonas* strains was higher than the pathogens (*p* < 0.05). As shown in Figure 1, comparing *Pseudomonas* strains revealed that the highest number of biofilm cells on the stainless steel surfaces were from *P. simiae* 77a (5.89 ± 0.51 log CFU/cm^2^), and the lowest number was from *P. cedrina* 69a (3.66 ± 0.47 CFU/cm^2^). Among the pathogens, the highest and lowest number of biofilm cells were from *S. aureus* (2.87 ± 0.43 CFU/cm^2^) and *E. coli* O157:H7 (2.08 ± 0.31 CFU/cm^2^), respectively.

### Dual-species biofilms

#### *Pseudomonas fluorescens* group strains and *Staphylococcus aureus*

**Figure 2:**
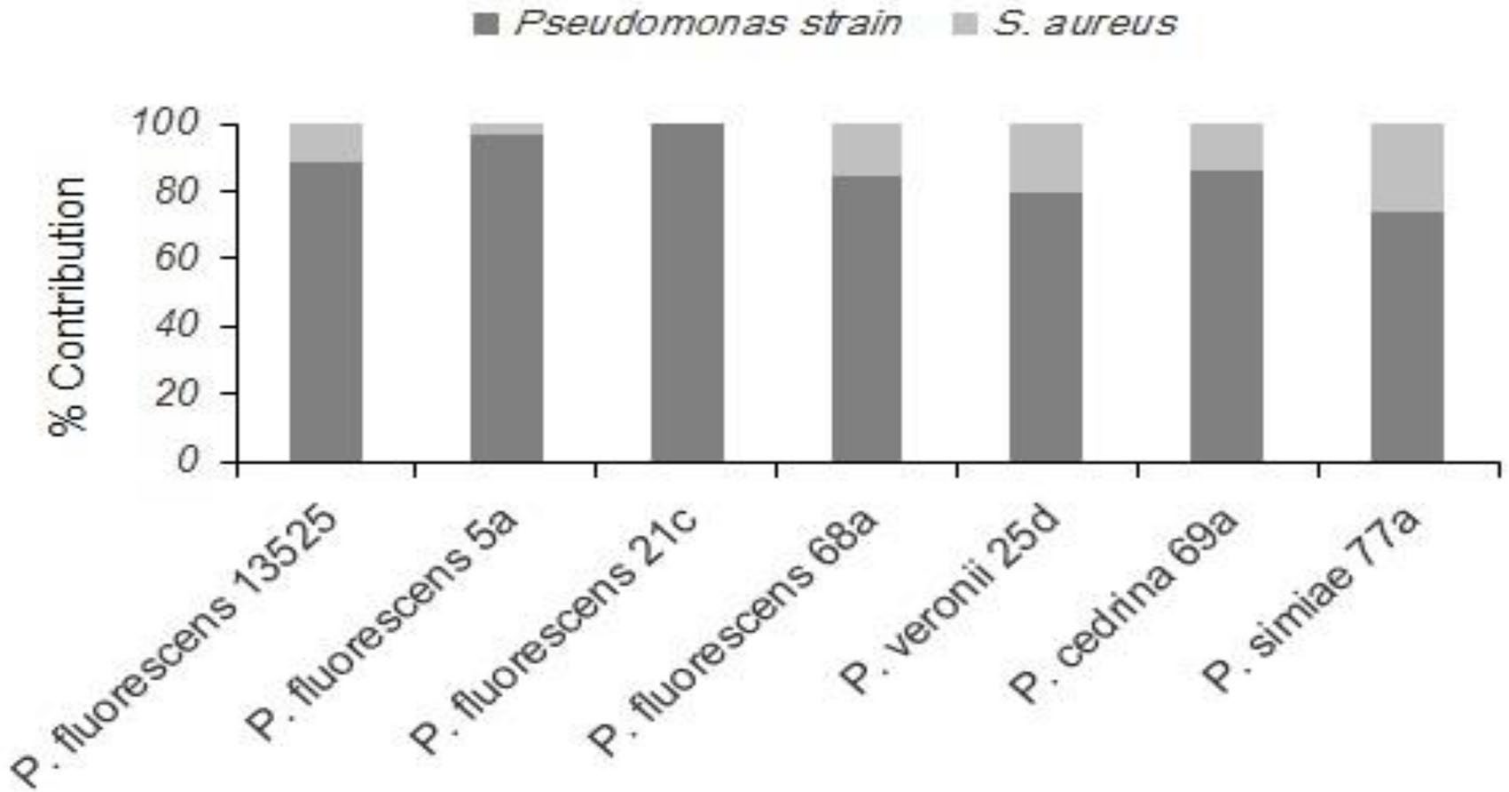
Contributions of different *P. fluorescens* group strains and *S. aureus* to dual-species biofilms on stainless steel surfaces under the CI scenario.

**Figure 3:**
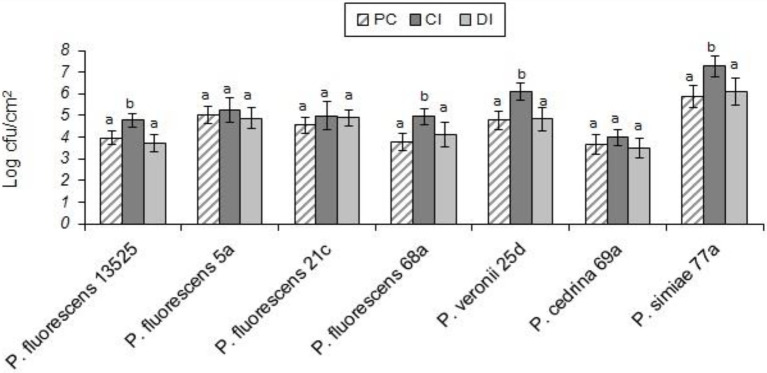
Total number of biofilm cells in mono- and dual-species biofilms of the seven *P. fluorescens* group strains with *S. aureus* on stainless steel surfaces. PC: pure culture biofilms; CI: dual-species biofilms under the CI scenario; DI: dual-species biofilms under the DI scenario. For each *P. fluorescens* strain, different letters indicate significant differences in the total number of biofilm cells.

Evaluating the formation of dual-species biofilm by different *P. fluorescens* group strains and *S. aureus* showed differences between *P. fluorescens* group strains within each scenario, and also between scenarios. In the CI scenario, *S. aureus* was found in six of the seven biofilms, with varying degrees of contribution. As shown in Figure 2, the highest contributions were observed with *P. simiae* 77a (24.3%) and *P. veronii* 25d (20.4%), while the lowest was observed with *P. fluorescens* 5a (3.6%). In this scenario, *S. aureus* did not contribute to the biofilm formed by *P. fluorescens* 21c. Additionally, there were significantly more dual-species biofilm cells with *P. fluorescens* ATCC 13525, *P. fluorescens* 68a, *P. veronii* 25d, and *P. simiae* 77a than in their single-species biofilms (*p* < 0.05; Figure 3). In contrast, *S. aureus* did not contribute to the structure of any biofilms in the DI scenario. Biofilms formed in pure cultures of *P. fluorescens* group strains did not differ significantly from those formed in the DI scenario in terms of the number of biofilm cells (*p* > 0.05; Figure 3).

#### *Pseudomonas fluorescens* group strains and *Bacillus cereus*

**Figure 4:**
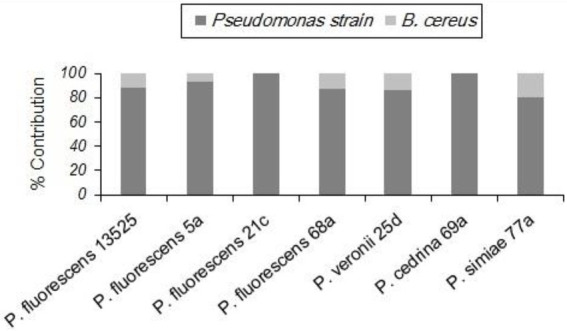
Contributions of different *P. fluorescens* group strains and *B. cereus* to dual-species biofilms on stainless steel surfaces in CI scenario.

**Figure 5:**
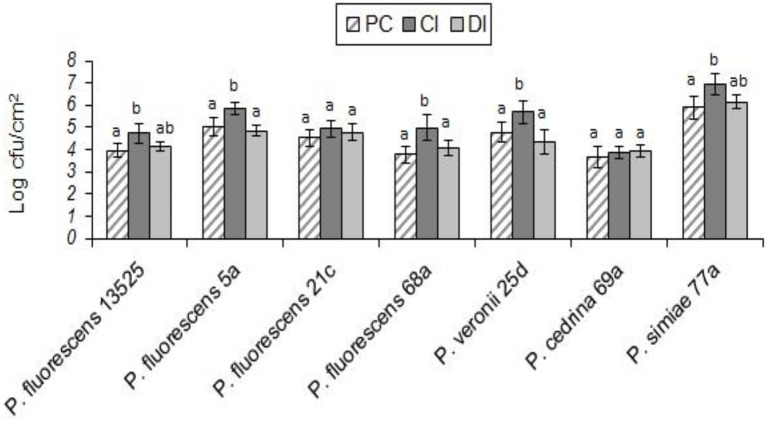
Total number of biofilm cells in mono- and dual-species biofilms of the seven *P. fluorescens* group strains with *B. cereus* on stainless steel surfaces. PC: pure culture biofilms; CI: dual-species biofilms under the CI scenario; DI: dual-species biofilms under the DI scenario. For each *P. fluorescens* strain, different letters indicate significant differences in the total number of biofilm cells.

As with *S. aureus*, *B. cereus* did not contribute to the biofilm formation in the DI scenario, and the total number of biofilm cells did not differ significantly from those formed in pure cultures of *P. fluorescens* group strains. In contrast, *B. cereus* contributed to five out of seven biofilms in the CI scenario. As shown in Figure 4, the highest contributions were observed with *P. simiae* 77a (19.4%) and *P. veronii* 25d (14.3%), while the lowest was observed with *P. fluorescens* 5a (6.9%); *B. cereus* did not contribute to the biofilm formed by *P. fluorescens* 21c and *P. cedrina* 69a. The total number of biofilm cells in dual-species biofilms with *B. cereus* and *P. fluorescens* ATCC 13525, *P. fluorescens* 5a, *P. fluorescens* 68a, *P. veronii* 25d and *P. simiae* 77a were significantly higher than those in the pure cultures of *P. fluorescens* group strains (*p* < 0.05; Figure 5).

#### *Pseudomonas fluorescens* group strains and *Escherichia coli* O157:H7

**Figure 6:**
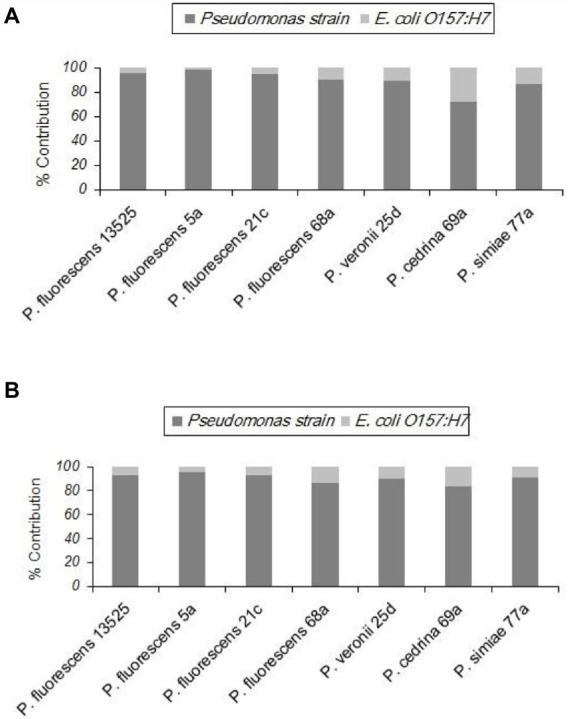
Contributions of different *P. fluorescens* group strains and *E. coli* O157:H7 to dual-species biofilms on stainless steel surfaces under CI **(A)** and DI **(B)** scenarios.

Unlike the two gram-positive bacteria that did not contribute to the biofilm structure in the DI scenario, *E. coli* O157:H7 contributed to biofilm formation with all seven *P. fluorescens* group strains in both CI and DI scenarios, to varying degrees. As shown in Figure 6, the highest percent contribution was found with *P. cedrina* 69a (27.8 and 16.7% in the CI and DI scenarios, respectively), and the lowest was found with *P. fluorescens* 5a (2.1 and 4.7% in the CI and DI scenarios, respectively).

**Figure 7:**
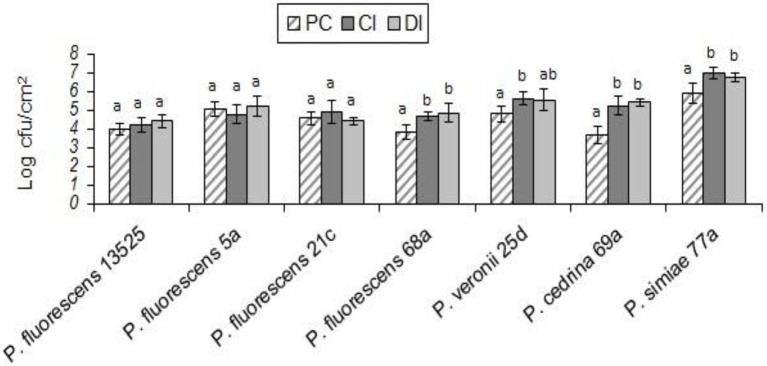
Total number of biofilm cells in mono-and dual-species biofilms of the seven *P. fluorescens* group strains with *E. coli* O157:H7 on stainless steel surfaces. PC: pure culture biofilms; CI: dual-species biofilms under the CI scenario; DI: dual-species biofilms under the DI scenario. For each *P. fluorescens* strain, different letters indicate significant differences in the total number of biofilm cells.

In both scenarios, the total number of biofilm cells in dual-species biofilms with *E. coli* O157:H7 and *P. fluorescens* 68a, *P. cedrina* 69a and *P. simiae* 77a were significantly higher than those in pure cultures of *P. fluorescens* group strains (Figure 7; *p* < 0.05). For *P. veronii* 25d, the total number of biofilm cells in dual-species biofilms was significantly higher than in the pure culture (p < 0.05), but only in the CI scenario. No significant differences were observed between CI and DI scenarios in terms of the number of biofilm cells in dual-species biofilms of the seven *P. fluorescens* group strains with *E. coli* O157:H7 (*p* > 0.05; Figure 7).

#### *Pseudomonas fluorescens* group strains and *Salmonella* Typhimurium

**Figure 8:**
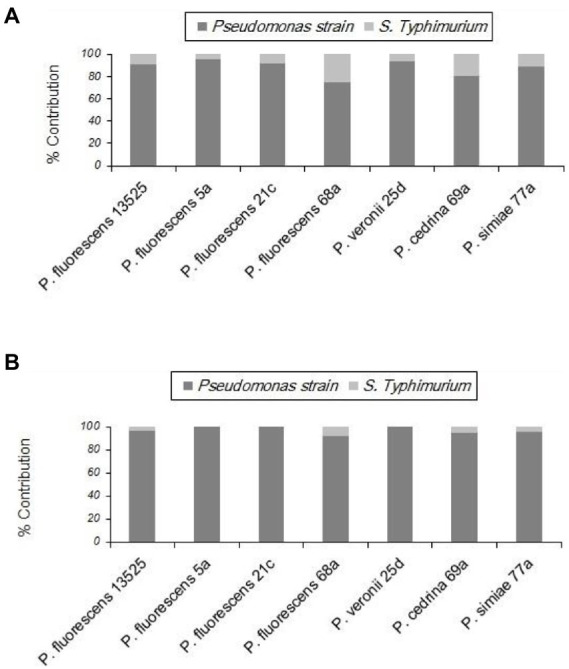
Contributions of different *P. fluorescens* strain groups and *S.* Typhimurium to dual-species biofilms on stainless steel surfaces under CI **(A)** and DI **(B)** scenarios.

With *S.* Typhimurium, the situation was somewhat different from that of the other Gram-negative bacteria tested (*E. coli* O157:H7). *S.* Typhimurium contributed to biofilm formation with all seven *P. fluorescens* group strains in the CI scenario, to varying degrees. As shown in Figure 8A, the highest contribution was found with *P. fluorescens* 68a (25.2%), and the lowest was with *P. fluorescens* 5a (4.6%). However, in the DI scenario, *S.* Typhimurium did not contribute to the biofilm formed by *P. fluorescens* 5a, *P. fluorescens* 21c, or *P. veronii* 25d. It did contribute to dual-species biofilms with other *P. fluorescens* group strains, but to a lesser extent than in the CI scenario (Figure 8B).

**Figure 9:**
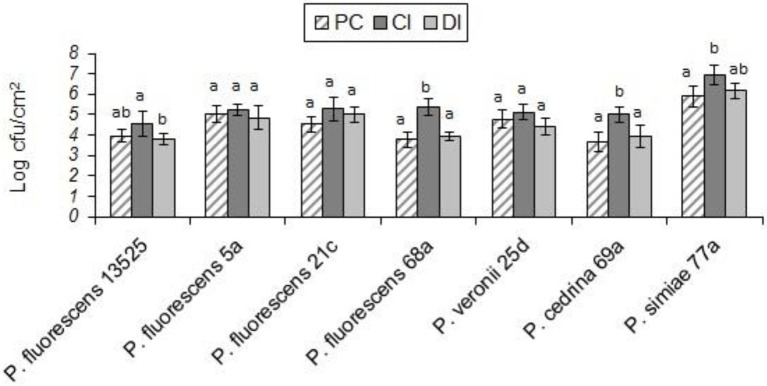
Total number of biofilm cells in mono- and dual-species biofilms of the seven *P. fluorescens* group strains with *S. typhimurium* on stainless steel surfaces. PC: pure culture biofilms; CI: dual-species biofilms under the CI scenario; DI: dual-species biofilms under the DI scenario. For each *P. fluorescens* strain, different letters indicate significant differences in the total number of biofilm cells.

As shown in Figure 9, in both scenarios the total numbers of biofilm cells in dual-species biofilms of *P. fluorescens* 5a, *P. fluorescens* 21c and *P. veronii* 25d were not significantly different from those in pure cultures of *P. fluorescens* group strains (p > 0.05). In contrast, the total number of biofilm cells in dual-species biofilms with *S.* Typhimurium and *P. fluorescens* 68a and *P. cedrina* 69a in the CI scenario were significantly higher than those in the DI scenario, and in pure cultures (*p* < 0.05). Furthermore, the total number of biofilm cells in dual-species biofilms with *S.* Typhimurium and *P. fluorescens* 13,525 and *P. simiae* 77a in the CI scenario were significantly higher than that in the DI scenario, and in the pure culture, respectively (p < 0.05).

## Discussion

*P. fluorescens* group strains are among the bacteria most frequently isolated from surfaces in the food industry, and are characterized as quick and thick biofilm producers on various surfaces (Simoes et al., 2008; Mann and Wozniak, 2012; Marchand et al., 2012; Puga et al., 2016; Zarei et al., 2020). Results of the present study re-confirmed the high ability of these bacteria to produce biofilm on stainless steel surfaces. In general, at 7°C these bacteria produced more biofilm than the pathogenic strains tested, however the *P. fluorescens* strains differed significantly in their ability to produce biofilms on stainless steel surfaces in UHT milk. Previous research has highlighted strain diversity of the *P. fluorescens* group isolated from dairy products and raw milk in terms of blue pigment production and lipoproteolytic activity (Chierici et al., 2016; Longhi et al., 2022). Other differences in the proteolytic activity of *P. fluorescence* group strains have also been reported previously (Zarei et al., 2020).

The presence of spoilage and pathogenic bacteria in milk and dairy products is a worldwide problem that not only leads to shelf-life reduction and alteration of organoleptic properties, but is also related to many disease outbreaks. The coexistence and interactions between foodborne pathogens and resident background microbiota are likely to occur on the surface of milk tanks and other dairy processing equipment between sessile cells, particularly in biofilms. It has been well documented that most naturally occurring biofilms are composed of multiple bacterial species (Yannarell et al., 2019). Hence, in terms of pathogen densities in dual-or multi-species biofilms, interactions with background microbiota strains can have neutral, positive, or antagonistic effects (Langsrud et al., 2016; Møretrø and Langsrud, 2017; Heir et al., 2018). These interactions can protect bacteria from environmental stresses, and can also influence the growth and survival of the individual members of these microbial consortia (Røder et al., 2015; Sanchez-Vizuete et al., 2015; Møretrø and Langsrud, 2017; Papaioannou et al., 2018).

In the present study, the interactions between *P. fluorescens* group bacterial strains and *S. aureus*, *B. cereus*, *E. coli* O157:H7, and *S.* Typhimurium during dual-species biofilm formation on stainless steel surfaces in UHT milk were evaluated. Two scenarios (concurrent inoculation and delayed inoculation of pathogens) were examined to better understand the possibility of dual-species biofilm formation. Given that all experiments in this study were performed at 7°C, the predominance of the *Pseudomonas* population in biofilms in both scenarios was expected.

In dual-species biofilms with *S. aureus*, this pathogen was found in six of the seven biofilms, with varying degrees of contributions in the CI scenario, which revealed differences between various strains within the *P. fluorescens* group. Moreover, our findings indicated that although *S. aureus* could co-produce biofilms with *P. fluorescens* group bacterial strains, it could not be incorporated into the pre-formed biofilms. Almost the same results were observed for another gram-positive bacterium used in this study – *B. cereus*. This pathogen was found in five of the seven biofilms, also with varying degrees of contributions in the CI scenario, while it did not contribute to biofilm formation in the DI scenario. The metabolites secreted around the biofilm matrix of *P. fluorescens* group strains may have prevented these two gram-positive pathogens from entering the pre-formed biofilms. Insufficient places to attach to the surface can be another explanation for the lack of contribution of these two gram-positive bacteria to the biofilm formation in the DI scenario (Balaure and Grumezescu, 2020). Evidently, biofilm formation in the CI scenario was not hampered by this challenge, leading to dual-species biofilms. Simoes et al. (2008) demonstrated that dual-species biofilms of *P. fluorescens* and *B. cereus* were significantly more metabolically active than *P. fluorescens* mono-species biofilms. Furthermore, Davies and Marques (2011) found that the fatty acid cis-2-decenoic acid produced by *Pseudomonas* group strains yielded dispersion of biofilms formed by *S. aureus*.

In contrast to the two gram-positive bacteria, *E. coli* O157:H7 and *S.* Typhimurium formed dual-species biofilms with all seven strains of the *P. fluorescens* group in the CI scenario, and with seven (*E. coli* O157:H7) and four (*S.* Typhimurium) strains in the DI scenario. Nevertheless, for both pathogens, significant differences were observed between *P. fluorescens* group strains in both scenarios, indicating strong and weak competitor strains within the *P. fluorescens* group. It has been previously reported that the biofilm formation of *E. coli* O157:H7 increased in the presence of background microbiota of meat (Dourou et al., 2011). This might explain why *E. coli* O157:H7 had a higher percent contribution to biofilm formation in the CI scenario than the DI scenario.

Overall, this study provides the first demonstration of differences between strains of the *P. fluorescens* group in terms of the formation of mono-or dual-species biofilms with pathogenic bacteria. Moreover, the ability to produce dual-species biofilms with pathogenic bacteria depends on whether the pathogens form the biofilm simultaneously with the *P. fluorescens* group strains or whether *P. fluorescens* group strains have already formed a biofilm. Such differences between strains and the inoculation time makes comparison of the results difficult, but also highlights the complexity of bacterial interactions involving the strains of the *P. fluorescens* group that requires further understanding for improved control of biofilms in the dairy industry. This study also contributes to the understanding of the role of different strains of the *P. fluorescens* group in the ability of four important foodborne pathogens to establish, survive and persist in dairy processing premises.

## Data availability statement

The original contributions presented in the study are included in the article/supplementary material, further inquiries can be directed to the corresponding authors.

## Author contributions

MZ designed the research. AY and SR performed the experiments. MZ and PS performed data analysis. MZ and AY prepared the figures. MZ wrote the manuscript, with contributions from AY and PS. All authors contributed to the article and approved the submitted version.

## Funding

This study was supported by the research grant provided by Shahid Chamran University of Ahvaz (SCU.VF99.245) and open access funded by Helsinki University Library.

## Conflict of interest

The authors declare that the research was conducted in the absence of any commercial or financial relationships that could be construed as a potential conflict of interest.

## Publisher’s note

All claims expressed in this article are solely those of the authors and do not necessarily represent those of their affiliated organizations, or those of the publisher, the editors and the reviewers. Any product that may be evaluated in this article, or claim that may be made by its manufacturer, is not guaranteed or endorsed by the publisher.
